# Validation of the Oxford WebQ Online 24-Hour Dietary Questionnaire Using Biomarkers

**DOI:** 10.1093/aje/kwz165

**Published:** 2019-07-18

**Authors:** Darren C Greenwood, Laura J Hardie, Gary S Frost, Nisreen A Alwan, Kathryn E Bradbury, Michelle Carter, Paul Elliott, Charlotte E L Evans, Heather E Ford, Neil Hancock, Timothy J Key, Bette Liu, Michelle A Morris, Umme Z Mulla, Katerina Petropoulou, Gregory D M Potter, Elio Riboli, Heather Young, Petra A Wark, Janet E Cade

**Affiliations:** 1 School of Medicine, University of Leeds, Leeds, United Kingdom; 2 Leeds Institute for Data Analytics, University of Leeds, Leeds, United Kingdom; 3 Nutrition and Dietetic Research Group, Division of Diabetes, Endocrinology and Metabolism, Faculty of Medicine, Imperial College London, London, United Kingdom; 4 Academic Unit of Primary Care and Population Sciences, Faculty of Medicine, University of Southampton, Southampton, United Kingdom; 5 NIHR Southampton Biomedical Research Centre, University of Southampton and University Hospital Southampton NHS Foundation Trust, Southampton, United Kingdom; 6 Cancer Epidemiology Unit, Nuffield Department of Population Health, University of Oxford, Oxford, United Kingdom; 7 National Institute for Health Innovation, University of Auckland, Auckland, New Zealand; 8 Nutritional Epidemiology Group, School of Food Science and Nutrition, University of Leeds, Leeds, United Kingdom; 9 MRC-PHE Centre for Environment and Health, Department of Epidemiology and Biostatistics, School of Public Health, Faculty of Medicine, Imperial College London, London, United Kingdom; 10 NIHR Imperial Biomedical Research Centre, Imperial College London, London, United Kingdom; 11 School of Public Health and Community Medicine, University of New South Wales, Sydney, New South Wales, Australia; 12 Global eHealth Unit, Department of Primary Care and Public Health, School of Public Health, Imperial College London, London, United Kingdom; 13 Centre for Innovative Research Across the Life Course, Faculty of Health and Life Sciences, Coventry University, Coventry, United Kingdom

**Keywords:** dietary assessment, diet questionnaires, Million Women Study, nutrition assessment, recall, recovery biomarkers, UK Biobank, validation

## Abstract

The Oxford WebQ is an online 24-hour dietary questionnaire that is appropriate for repeated administration in large-scale prospective studies, including the UK Biobank study and the Million Women Study. We compared the performance of the Oxford WebQ and a traditional interviewer-administered multiple-pass 24-hour dietary recall against biomarkers for protein, potassium, and total sugar intake and total energy expenditure estimated by accelerometry. We recruited 160 participants in London, United Kingdom, between 2014 and 2016 and measured their biomarker levels at 3 nonconsecutive time points. The measurement error model simultaneously compared all 3 methods. Attenuation factors for protein, potassium, total sugar, and total energy intakes estimated as the mean of 2 applications of the Oxford WebQ were 0.37, 0.42, 0.45, and 0.31, respectively, with performance improving incrementally for the mean of more measures. Correlation between the mean value from 2 Oxford WebQs and estimated true intakes, reflecting attenuation when intake is categorized or ranked, was 0.47, 0.39, 0.40, and 0.38, respectively, also improving with repeated administration. These correlations were similar to those of the more administratively burdensome interviewer-based recall. Using objective biomarkers as the standard, the Oxford WebQ performs well across key nutrients in comparison with more administratively burdensome interviewer-based 24-hour recalls. Attenuation improves when the average value is taken over repeated administrations, reducing measurement error bias in assessment of diet-disease associations.

Dietary intakes estimated from self-reported dietary assessments are prone to measurement error, introducing potentially substantial bias and loss of statistical power (1, 2). It is therefore important to calibrate self-reported intakes against objective biomarkers, where measurement errors can be assumed to be independent (3, 4). Most cohort studies have used food frequency questionnaires (FFQs) designed to assess diet over the long term, but short-term recalls may have less bias from measurement error (5–7), other than for episodically consumed foods. Repeated application of short-term recalls may offer longer-term coverage, but the process is administratively burdensome. Online dietary assessment offers repeated administration with reduced administrative costs (8), but to facilitate this, the assessment instrument must be convenient for the participant to use (9, 10).

The Oxford WebQ is an online dietary questionnaire covering the previous day’s intake (11), developed to provide an easy-to-complete dietary assessment appropriate for repeated use in large-scale prospective studies. It is currently being used in the UK Biobank (12–14) and the Million Women Study (15, 16).

The Oxford WebQ has previously been shown to provide results similar to those derived from an interviewer-administered self-report 24-hour dietary recall, but it is quicker to complete (17, 18). However, the comparison tool was itself a self-report instrument, providing an inadequate basis for validation, because self-report tools are prone to correlated person-specific biases (19–21). These biases may differ according to personal characteristics such as age, sex, or body mass index (BMI; weight (kg)/height (m)^2^).

We therefore aimed to provide the first validation of the Oxford WebQ tool against established recovery and predictive nutritional biomarkers and a reference measure of energy expenditure free from these person-specific biases. In doing so, we present the degree to which diet-disease relationships assessed using the Oxford WebQ are attenuated and the extent to which statistical power to detect these comparisons is reduced even in large-scale studies such as the UK Biobank study and the Million Women Study.

## METHODS

### Recruitment

Participants were enrolled in a United Kingdom study designed to validate both the Oxford WebQ dietary assessment tool and the myfood24 dietary assessment tool (22) against nutritional biomarkers, comparing these with a standard interviewer-based multiple-pass 24-hour dietary recall, hereafter called the multiple-pass recall (MPR) (23). Eligibility criteria were aimed at recruiting participants who were broadly representative of the adult general population. Participants were eligible for the study if they were between 18 and 65 years of age and were maintaining a stable weight, confirmed by no substantive weight loss or weight gain over the course of the study (>5% weight change from first clinic appointment). Further criteria included regular access to high-speed Internet service, use of a telephone, and ability to speak and read English so the participant could complete the online questionnaires and 24-hour recalls. Participants had to be willing to visit the Clinical Research Facility at Hammersmith Hospital, London, United Kingdom (Imperial College Healthcare NHS Trust), to provide blood and urine samples.

Participants were identified between 2014 and 2016 through a multidisciplinary network of primary-care professionals and practices, the North West London Primary Care Research Network, and persons known to the Clinical Research Facility who had previously expressed an interest in participating in research projects. Participants were also identified from a list of local addresses provided by the post office. Participants were not a subsample of the UK Biobank subjects but an independent sample designed to be of a similar age and sex distribution as the UK Biobank subjects. Upon completion of the study, participants were provided with modest financial reimbursement for their time. The recruitment target was 200 participants with complete information collected (see Web Appendix 1, available at https://academic.oup.com/aje).

### Overview of study design

**Figure 1.**
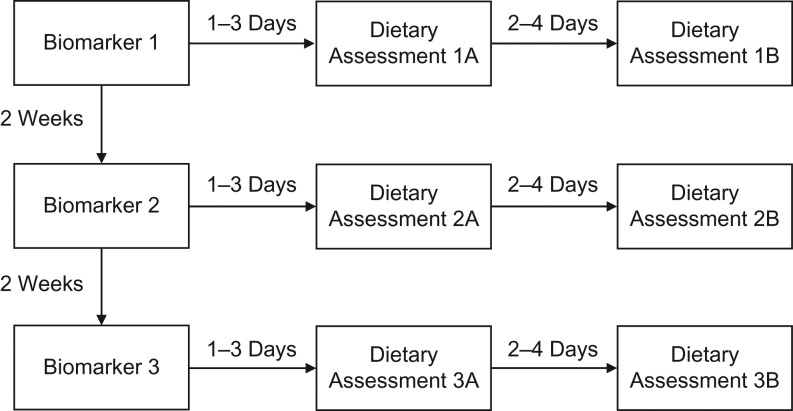
Design of a validation study of the Oxford WebQ, an online 24-hour dietary questionnaire, United Kingdom, 2014–2016. Each 24-hour dietary assessment (the Oxford WebQ online tool and the interviewer-based multiple-pass 24-hour recall, in random order) and selected reference measurements (recovery biomarkers, predictive biomarkers, and total energy expenditure) were completed on 3 different occasions separated by approximately 2 weeks. On each occasion, the reference measurement was followed 1–3 days later by the first dietary assessment, which was followed approximately 2–4 days later by the second dietary assessment.

Each participant provided 3 sets of urine samples and accelerometry data for reference measures (recovery biomarkers, predictive biomarkers, and total energy expenditure (TEE)) and completed 3 MPRs and 3 Oxford WebQ online dietary questionnaires, all spread over a 5-week period. This data collection was achieved in 3 separate cycles, carried out 2 weeks apart (Figure 1). At the start of each cycle, participants provided urine and accelerometry for the set of reference measures, followed by a dietary assessment 1–3 days later and another dietary assessment 2–4 days after that. The order of the dietary assessments within each cycle was allocated by simple randomization, to reduce order effects. Each of the assessments is described in detail below.

### Biomarkers

Participants provided 24-hour urine samples, discarding the first morning void and then collecting every subsequent urine specimen for the remaining 24 hours, ending with the last specimen the following morning. Urine specimens were then returned to the clinic on the day that collection ended. Urine volumes were recorded, and urine was then aliquoted into separate 50-mL aliquots before being stored at −20°C and transported to the Molecular Epidemiology Unit at the University of Leeds (Leeds, United Kingdom). The Kjeldahl method (24) was used to measure the total urinary nitrogen content of the samples. Participants took 3 80-mg 4-aminobenzoic acid (PABA) tablets with meals during the course of the 24-hour urine sampling period for confirmation of sample completeness (25). The concentration of PABA in the urine was measured using high-performance liquid chromatography. We considered 93% PABA to indicate complete urine collection over the 24-hour period, but 85%–110% was permissible, consistent with previous research (25).

Protein intake was estimated on the basis of the assumption that 81% of nitrogen is excreted within 24 hours (26). Potassium intakes were estimated from the amount excreted in the urine, as measured by the Clinical Biochemistry Department at the Leeds Teaching Hospitals NHS Trust using an ADVIA 2400 Clinical Chemistry System (Siemens AG, Munich, Germany) with ion-selective electrode detection. We assumed that 80% of potassium intake is excreted in the urine (27).

Urinary concentrations of fructose and sucrose were measured using a Sucrose/D-Glucose/D-Fructose assay (Boehringer Mannheim/R-Biopharm AG, Darmstadt, Germany) scaled down to a microplate format. Daily excretion of urinary sucrose and fructose was then estimated on the basis of total urine volume collected over 24 hours. The predicted intake of total sugars for each individual, allowing for age and sex, was then estimated using a calibration equation derived from previous feeding studies comprising 30 days’ intervention in a metabolic suite under controlled conditions (28, 29).

### Total energy expenditure

Resting energy expenditure was measured using open-loop indirect calorimetry (Gas Exchange Monitor; GEM Nutrition, Daresbury, United Kingdom), assessed at the research facility when participants came for their clinic visit. The calorimeter was calibrated, and volunteers lay in a semirecumbent position. Following stabilization of measurements, oxygen consumption (volume of oxygen (VO_2_)) and carbon dioxide production (volume of carbon dioxide (VCO_2_)) were recorded every minute for 15 minutes. The mean values of the last 10 sets of measurements were used to estimate resting energy expenditure (30). Activity energy expenditure was also estimated, using 3-plane accelerometry, by means of a SenseWear armband mini-accelerometer (BodyMedia Inc., Pittsburgh, Pennsylvania). This was worn for 24 hours on the left upper arm on one of the days before each clinic visit. The thermic effect of food was assumed to be 10% of TEE (31). TEE was estimated by summing resting energy expenditure, activity energy expenditure, and the thermic effect of food, with estimated TEE indicating total energy intake, provided that the participant remained in energy balance. This method has previously demonstrated close agreement with energy expenditure estimated using doubly labeled water (32). TEE estimates for participants with more than a 5% weight change over the course of the study were excluded. Within-person variability was taken into account in statistical analysis for all repeated measures.

### Oxford WebQ online dietary questionnaire

The development of the Oxford WebQ online dietary questionnaire has been fully described elsewhere (11, 17, 18). Briefly, the tool was designed as a Web-based dietary questionnaire that was easy to use by both participants and researchers in large-scale observational studies, through extensive piloting and iterative improvement. The Oxford WebQ presents participants with 21 broad food groups, with options then expanding to offer over 200 commonly consumed foods and drinks. The participants are prompted to select the amount consumed over the previous 24 hours, mostly from predefined categories offered to them. To facilitate large-scale automatic coding of nutrient information, use of free-text boxes is minimized. Upon completion of the tool, the participants are presented with a summary page of all the food and drink items they reported consuming, together with the amounts reported, and are asked to make any necessary amendments. Completed questionnaires are coded automatically through multiplication of amounts consumed by the nutrient contents specified in standard United Kingdom food composition tables (33), producing a profile of the intake of 21 separate nutrients, without any additional intervention required by nutritionists.

### Interviewer-administered 24-hour recall

To facilitate comparison of the Oxford WebQ with an equivalent interviewer-administered tool, participants also completed an MPR, which was conducted over the telephone by a trained researcher using a prompt sheet based on the 5-step multiple-pass method (34). Participants were asked to provide details on cooking methods, brand names, and portion sizes. Nutrient intake was estimated using Dietplan6.7 software (Forestfield Software, Horsham, United Kingdom), based on the same food composition tables as the Oxford WebQ (33). Trained researchers matched the food and drink items recorded to the food composition tables and applied the portion sizes using a standard operating protocol, which is described fully elsewhere (35).

### Statistical analysis

Urine samples with 2 or more voids missed during the 24-hour period were excluded. Apart from this, the main analyses included all participants (36). The robustness of urinary biomarker results to completeness of the urine samples was assessed by conducting a sensitivity analysis including only participants who had complete PABA recovery (85%–110%) or whose PABA recovery was 50%–85% with their urinary nitrogen and potassium rescaled to the 93% PABA recovery expected for complete recovery, consistent with previous research (37).

We present results for both nutrients and nutrient densities. The densities are defined as the ratio of nutrient intake (g) to energy intake (MJ) measured by the same dietary assessment tool to represent energy-adjusted quantities derived from the tool. All nutrient intake and nutrient density data were log-transformed prior to statistical analysis to better approximate normal distributions. All statistical analyses were performed in Stata, version 14.2 (StataCorp LLC, College Station, Texas) (38).

### Measurement error models

A measurement error structure similar to that used by the Observing Protein and Energy Nutrition (OPEN) Study and the European Prospective Investigation Into Cancer and Nutrition (EPIC)–Norfolk (20, 39) was assumed, including linear associations between the longer-term true intake and both the biomarkers and self-reported intakes. We assumed person-specific systematic biases for both self-report tools, which were assumed to be correlated. We also assumed a systematic bias related to level of intake.

Our measurement error model follows that proposed by Kipnis et al. (19, 39). For Oxford WebQ estimate *Q*_*ij*_, interviewer-based MPR *F*_*ij*_, and biomarker *M*_*ij*_ on person *i* at occasion *j*,

$$Q_{ij}=\mu_{Qj}+\beta_{Q0}+\beta_{Q1}T_{i}+r_{i}+\varepsilon_{ij},$$

$$F_{ij}=\mu_{Fj}+\beta_{F0}+\beta_{F1}T_{i}+s_{i}+u_{ij}\text{,}$$

and

$$M_{ij}=\mu_{Mj}+T_{i}+v_{ij},$$

where *T*_*i*_ is the true intake for individual *i*; μ_*Qj*_ and μ_*Fj*_ represent possible drift over time between measures; β_*Q*0_, β_*Q*1_, β_*F*0_, and β_*F*1_ are biases, where β_*Q*0_ and β_*F*0_ are additive components associated with each tool and β_*Q*1_ and β_*F*1_ are multiplicative components; and *r*_*i*_ and *s*_*i*_ model the person-specific biases for each tool. We allow these person-specific biases to be correlated with ρ(*r*, *s*) ≠ 0, because the same mechanisms may be influencing both *r*_*i*_ and *s*_*i*_. We assume independent within-person errors ε_*ij*_ and *u*_*ij*_ that follow normal distributions with mean 0 and variances σ_ε_^2^ and σ_*u*_^2^, respectively.

We assume that there is no person-specific bias associated with biomarker *M*_*ij*_ and that within-person error *v*_*ij*_ follows a normal distribution with mean 0 and variance σ_*v*_^2^ and is independent of the true intake and other error components. For analyses assessing estimated intake from the Oxford WebQ based on the average of *k* serial measurements, variance σ_ε_^2^ is replaced by σ_ε_^2^/*k*.

We assume that correlation between biomarkers and dietary assessment measures does not vary by proximity in time because, for each cycle, the biomarker measure is completed before the dietary assessment day, and the gap between the final dietary assessment of a previous cycle and the biomarker collection for the next is short. Subsequent exploration of the observed correlation structure was consistent with this assumption (data not shown).

We assume that associations between urinary sucrose/fructose excretion and total sugar intake were similar to those in previously published feeding studies (28, 29), allowing us to apply calibration equations derived from those studies:

$${M^{*}}_{ij}=M_{ij}-1.67-0.02S_{i}+0.71A_{i},$$

where M^*^_ij_ is the calibrated biomarker value, *M*_*ij*_ is the observed biomarker value, *S*_*i*_ is 0 for men and 1 for women, and *A*_*i*_ is log-transformed age. M^*^_ij_ was then used in place of *M*_*ij*_ in the measurement error model defined above. Participants in the feeding study from which this calibration equation was derived were healthy adults aged 23–66 years (28), similar to the OPEN study population of healthy adults (ages 40–69 years) (29) and the UK Biobank population (ages 40–69 years) (12).

### Model-fitting

The measurement error models were fitted as structural equation models using maximum likelihood estimation, assuming that any missing data points were missing at random. Results are presented as attenuation factors indicating the extent to which estimated diet-disease associations are diluted using the Oxford WebQ. Attenuation factors closer to 1 indicate less bias in diet-disease estimates. The correlation between the Oxford WebQ and the latent variable in the structural equation model estimating true longer-term intake is also presented to indicate the amount of power lost in prospective studies using the Oxford WebQ. This correlation also represents the attenuation of log relative risks between equal-sized categories of intake estimated by the Oxford WebQ (5, 6, 20, 40). The Oxford WebQ is designed for repeated administration (17, 18), and in the UK Biobank, participants were invited to complete it on up to 5 separate occasions over a 16-month period, with the majority of responders completing it twice (41). We therefore present the predicted attenuation factors for the mean of several repeat administrations and derived from our estimated measurement model parameters. This takes the same approach as that used by Schatzkin et al. (40). We focus on the mean of 2 administrations to reflect current use in the UK Biobank.

The mean differences between the Oxford WebQ and recovery biomarkers (for protein, sodium, and potassium), the predictive biomarkers (sugars), and total energy intake (accelerometry) are presented. For each participant, this was based on the mean intake over the repeated cycles of the Oxford WebQ measures minus the mean over the repeated cycles of the biomarker and energy expenditure measures, back-transformed and expressed as a percentage. This is equivalent to the mean difference estimated by the Bland-Altman method of assessing agreement (42).

### Subgroup analyses

We repeated the analyses with stratification on sex, age (<40 vs. ≥40 years), and BMI (<25 vs. ≥25) to quantify the robustness of results to different participant characteristics and to explore the possible impacts of differences in person-specific biases.

### Ethics

The validation study was conducted according to the guidelines laid down in the Declaration of Helsinki. Full written informed consent was obtained from all participants included. The procedures of the validation study and associated documentation were reviewed and approved by the West London NHS Research Ethics Committee.

## RESULTS

In total, 225 potential participants were invited to undergo screening for eligibility. Of these, 7 persons (3%) were ineligible, 30 (13%) did not consent, and 27 (12%) subsequently withdrew consent. The remaining 161 persons (72%) completed Oxford WebQs and MPRs and provided samples for biomarkers on at least 1 occasion. After exclusion of missed voids, data were available for analysis from 160 participants, 152 (95%) of whom completed the Oxford WebQ after visit 1, 146 (91%) of whom completed it after visit 2, and 147 (92%) of whom completed it after visit 3; 130 participants (81%) completed all 3 WebQs. Of these, 434 WebQs (98%) were completed on weekdays. The median amount of time needed to complete the Oxford WebQ was 10 minutes (interquartile range, 10–15 minutes).

Demographic characteristics of the participants at recruitment are shown in Table 1. Participants appeared metabolically stable over the course of the study, with weights changing by more than 5% of weight at booking for only 6 (4%) participants. The energy expenditure readings of those participants were excluded from the analysis.

**Table 1.** Demographic and Lifestyle Characteristics of Participants in a Validation Study of the Oxford WebQ, by Sex, London, United Kingdom, 2014–2016^a^

Participant Characteristic	Men (*n* = 68)		Women (*n* = 92)	
	No.	%	No.	%
Age, years^b^	43 (16)		43 (16)	
Ethnicity				
White	50	74	65	71
Black	1	1	7	8
Asian	4	6	5	5
Mixed or other	12	18	12	13
Age at leaving educational system, years				
≤16	8	12	8	9
17–18	18	26	25	27
≥19	42	62	57	62
Smoking status				
Nonsmoker	52	77	72	78
Smoker	10	15	8	9
Weight, kg^b^	81 (13)		66 (12)	
Body mass index^c^				
<25	30	44	53	58
25–29	26	38	27	29
≥30	12	18	12	13

^a^ Numbers may not sum to totals because of missing data.

^b^ Values are expressed as mean (standard deviation).

^c^ Weight (kg)/height (m)^2^.

Estimated geometric mean intakes of protein, potassium, and total sugar and their associated nutrient densities are shown in Table 2 for the Oxford WebQ, the MPR, biomarkers, and reference tools relating to the first clinic visit. Estimated intakes from the Oxford WebQ were broadly similar to those from the MPR for all nutrients. Compared with biomarker measures, the Oxford WebQ overestimated protein and potassium intakes and underestimated total sugar intake, with estimated total energy intake less than the estimated TEE.

**Table 2.** Geometric Mean Values for Daily Protein, Potassium, and Total Sugar Intakes and Nutrient Density as Assessed by the Oxford WebQ, the Interviewer-Based Multiple-Pass 24-Hour Recall, and Biomarkers Related to the First Clinic Visit, London, United Kingdom, 2014–2016

Nutrient Measure	Oxford WebQ			Interviewer-Based 24-Hour Recall			Biomarker/Reference Tool		
	No. of Persons	Geometric Mean	95% CI	No. of Persons	Geometric Mean	95% CI	No. of Persons	Geometric Mean	95% CI
Nutrient intake, g									
Protein	152	85.0	79.3, 91.1	154	82.0	77.0, 87.4	152	70.2	65.7, 75.1
Potassium	152	3.3	3.1, 3.5	154	3.1	3.0, 3.3	152	2.1	2.0, 2.3
Total sugars	152	100.8	92.9, 109.4	154	88.9	82.0, 96.3	151	133.5	116.3, 153.2
Total energy expenditure, MJ	152	8.7	8.1, 9.2	154	8.5	8.1, 9.0	144	11.0	10.4, 11.5
Nutrient density^a^, g/MJ									
Protein	152	9.8	9.4, 10.2	154	9.6	9.2, 10.1	142	6.4	6.0, 6.9
Potassium	152	0.38	0.36, 0.40	154	0.37	0.35, 0.39	142	0.19	0.18, 0.21
Total sugars	152	11.6	10.9, 12.4	154	10.4	9.7, 11.2	141	12.1	10.4, 14.0

Abbreviation: CI, confidence interval.

^a^ Nutrient density for protein, potassium, and total sugars was expressed in grams per MJ of total energy intake.

Attenuation factors and correlations between the self-report tools and estimated true longer-term intake for a single application of the Oxford WebQ are shown in Table 3. For nutrient densities, attenuation factors were slightly higher and correlations were slightly lower than for unadjusted nutrient intakes. The full list of parameters estimated from the measurement models is shown in Web Table 1. Table 3 also shows the mean percentage difference between the self-report tools and the biomarker measures (Table 3). Mean percentage differences for the Oxford WebQ were similar to those for the MPR.

**Table 3.** Attenuation Factors for and Correlations Between Daily Dietary Intake Derived From Self-Report Tools and Estimated True Longer-Term Intake for a Single Application of the Oxford WebQ, London, United Kingdom, 2014–2016^a^

Nutrient Measure	Attenuation Factor	95% CI	Correlation With True Intake	95% CI	Mean Difference From Reference Tool, %	95% CI
Nutrient intake, g						
Protein						
Oxford WebQ	0.27	0.17, 0.36	0.40	0.27, 0.52	12	6, 19
MPR^b^	0.33	0.24, 0.43	0.46	0.36, 0.57	8	3, 14
Potassium						
Oxford WebQ	0.31	0.18, 0.44	0.34	0.20, 0.47	53	42, 64
MPR	0.35	0.22, 0.48	0.37	0.25, 0.49	47	37, 57
Total sugars						
Oxford WebQ	0.31	0.18, 0.44	0.33	0.20, 0.46	−25	−18, −32
MPR	0.16	0.01, 0.30	0.15	0.01, 0.30	−32	−25, −39
Total energy expenditure, MJ						
Oxford WebQ	0.22	0.12, 0.33	0.32	0.18, 0.46	−22	−17, −27
MPR	0.30	0.17, 0.42	0.36	0.22, 0.49	−22	−18, −27
Nutrient density^c^, g/MJ						
Protein						
Oxford WebQ	0.34	0.17, 0.51	0.29	0.16, 0.42	46	37, 55
MPR	0.26	0.10, 0.42	0.23	0.09, 0.36	41	32, 50
Potassium						
Oxford WebQ	0.33	0.12, 0.54	0.23	0.09, 0.37	99	83, 115
MPR	0.41	0.23, 0.59	0.33	0.19, 0.46	91	77, 106
Total sugars						
Oxford WebQ	0.32	0.15, 0.50	0.27	0.13, 0.41	−3	8, −12
MPR	0.16	−0.03, 0.35	0.13	−0.02, 0.28	−12	−1, −21

Abbreviations: CI, confidence interval; MPR, multiple-pass recall.

^a^ Data for all dietary measures and estimates were log-transformed.

^b^ Interviewer-based multiple-pass 24-hour dietary recall.

^c^ Nutrient density for protein, potassium, and total sugars was expressed in grams per MJ of total energy intake.

Using the mean of a series of 2, 3, 4, or 5 repeat administrations of the Oxford WebQ would substantially improve measurement properties (Table 4), with an associated reduction in bias. With 2 repeats of the tool, the most likely use within the UK Biobank as it currently stands, the attenuation factors and the correlation with true intake would improve markedly. With more repeats of the tool, the attenuation and the correlation with true intake would improve further.

**Table 4.** Attenuation Factors for and Correlations Between Daily Dietary Intake Derived From the Oxford WebQ Tool and Estimated True Longer-Term Intake for Repeat Administrations of the Oxford WebQ, London, United Kingdom, 2014–2016^a,b^

Nutrient Measure and No. of Repeat Administrations	Attenuation Factor	95% CI	Correlation With True Intake	95% CI
Nutrient intake, g				
Protein				
1	0.27	0.17, 0.36	0.40	0.27, 0.52
2	0.37	0.24, 0.49	0.47	0.33, 0.61
3	0.42	0.28, 0.56	0.50	0.35, 0.65
4	0.45	0.30, 0.60	0.52	0.37, 0.67
5	0.48	0.32, 0.64	0.53	0.38, 0.69
Potassium				
1	0.31	0.18, 0.44	0.34	0.20, 0.47
2	0.42	0.25, 0.60	0.39	0.24, 0.54
3	0.48	0.28, 0.68	0.42	0.26, 0.58
4	0.52	0.30, 0.73	0.44	0.27, 0.60
5	0.54	0.32, 0.77	0.45	0.28, 0.62
Total sugars				
1	0.31	0.18, 0.44	0.33	0.20, 0.46
2	0.45	0.26, 0.64	0.40	0.24, 0.55
3	0.53	0.31, 0.75	0.43	0.27, 0.60
4	0.59	0.34, 0.83	0.45	0.28, 0.62
5	0.62	0.36, 0.88	0.47	0.29, 0.64
Total energy expenditure, MJ				
1	0.22	0.12, 0.33	0.32	0.18, 0.46
2	0.31	0.16, 0.45	0.38	0.21, 0.54
3	0.35	0.19, 0.52	0.40	0.23, 0.58
4	0.38	0.20, 0.56	0.42	0.24, 0.60
5	0.40	0.21, 0.59	0.43	0.24, 0.62
Nutrient density^c^, g/MJ				
Protein				
1	0.34	0.17, 0.51	0.29	0.16, 0.42
2	0.51	0.27, 0.76	0.36	0.20, 0.51
3	0.62	0.32, 0.91	0.39	0.22, 0.56
4	0.69	0.36, 1.01	0.41	0.23, 0.59
5	0.73	0.38, 1.09	0.42	0.24, 0.61
Potassium				
1	0.33	0.12, 0.54	0.23	0.09, 0.37
2	0.48	0.17, 0.78	0.28	0.11, 0.44
3	0.57	0.21, 0.93	0.30	0.12, 0.48
4	0.62	0.23, 1.02	0.31	0.12, 0.50
5	0.66	0.24, 1.09	0.32	0.13, 0.52
Total sugars				
1	0.32	0.15, 0.50	0.27	0.13, 0.41
2	0.49	0.23, 0.75	0.33	0.16, 0.50
3	0.59	0.28, 0.91	0.36	0.18, 0.55
4	0.66	0.31, 1.01	0.38	0.19, 0.58
5	0.71	0.33, 1.09	0.40	0.20, 0.60

Abbreviation: CI, confidence interval.

^a^ Data for all dietary measures and estimates were log-transformed.

^b^ Estimates of measurement properties for the mean of repeated administrations of the tool are based on the parameters provided in Web Table 1, using the approach described by Schatzkin et al. (40).

^c^ Nutrient density for protein, potassium, and total sugars was expressed in grams per MJ of total energy intake.

When urinary biomarker concentrations were adjusted for completeness of urine samples and samples with PABA recovery less than 50% or more than 110% were excluded, attenuation factors and correlations were essentially unchanged (see Web Appendix 2).

There was some variation between subgroups defined by age group, sex, and BMI (Web Tables 2–4). Attenuation factors for protein, potassium, and sugar intake were higher in men than in women. Attenuation was worse in older people (age ≥40 years) than in younger people (age <40 years) for protein but similar between age groups for total sugars and better in older people for potassium and total energy intake. Participants with BMI ≥25 had attenuation broadly similar to that of people with BMI <25, but with generally greater disparities for correlation with the truth.

## DISCUSSION

Our findings show that the Oxford WebQ dietary assessment tool being used in the UK Biobank and a sample of the Million Women Study has good measurement error properties, improving further when the mean of several measures is taken.

The Oxford WebQ tends to overestimate potassium intake and underestimate total sugar intake, but these figures were similar for the interviewer-administered MPR. Additionally, the Oxford WebQ is of broadly equivalent validity to the MPR in terms of attenuation of diet-disease associations. This held across 3 nutrients that could be measured by recovery biomarkers and other objective reference tools. For total sugars, the Oxford WebQ performed better than the interviewer-administered MPR. However, the Oxford WebQ is substantially quicker and cheaper to implement (17, 18).

The Oxford WebQ compares well to a recently validated online 24-hour recall used in the United Kingdom (23). The validity of the Oxford WebQ is also broadly similar to 24-hour recalls that have been validated in the United States (5, 6), though our finding that protein is overreported and potassium underreported in the United Kingdom contrasts with the underreporting of protein and unbiased reporting of potassium in the United States, on average. This may reflect the shorter length of assessment in our study compared with most US studies or different cultural perceptions of foods with high concentrations of those nutrients.

FFQs generally estimate diet over a longer time scale than the 24-hour period covered by the Oxford WebQ. Similarly, in common with 24-hour recalls, the Oxford WebQ cannot estimate past diet, while FFQs may be used for this purpose. However, repeated measures of the Web-based Oxford WebQ tool throughout follow-up, covering different seasonal intakes and reflecting dietary changes as the cohort ages, can provide an estimate of long-term diet in a more prospective manner. The correlations we found between the mean of 2–5 Oxford WebQ estimates and the truth were also better than those previously reported for FFQs (5, 6, 29), though no better for nutrient densities. The improvement in measurement properties upon repeat administration reflects how the Oxford WebQ is currently being used in the UK Biobank (18, 41). It is possible that a tool optimized for mobile phones could be used in a more prospective manner, further improving performance.

We have focused on use of the Oxford WebQ to estimate true longer-term diet and the extent to which measurement error in this estimated exposure could lead to attenuated estimates of the association with disease outcomes. This application of the tool for estimation of longer-term diet is the most relevant to large-scale cohort studies with long follow-up. Dietary exposures are often categorized to simplify presentation and because it is harder to estimate absolute intake precisely than to simply rank intakes from low to high. We therefore present the correlation between the Oxford WebQ intake and estimated true longer-term intake, which reflects the attenuation in diet-disease estimates based on ranked exposures. The Oxford WebQ generally performed slightly better according to this criterion. The Oxford WebQ performed well in comparison with other tools assessed using the same statistical methodology (21, 23).

In assessing the validity of the Oxford WebQ, we used objective biomarkers that are free from person-specific biases shared by self-report tools. Our validation was therefore more robust than validation using another self-report tool that may agree well partly because it shares this same bias. However, 45% of urine samples contained less than 85% PABA recovery, which could have led to underestimation of the agreement between self-reported diet and urinary biomarkers. Biomarker data were collected at clinic visits prior to completion of the dietary recalls, but participants were not informed of results, which minimized potential recall bias. Had biomarker collection coincided with dietary recall measures, there may have been greater agreement between them.

We did not use doubly labeled water to estimate TEE, which is a potential weakness in our study. In addition to estimated energy intake, this could also have affected nutrient density estimates and could partly explain why our results differed from those of previous studies which generally found that using densities improved measurements. However, use of activity monitor equipment provided an equally objective measure, which we used instead. It is a potential weakness that activity monitors were only worn for 1 day during each cycle, but within-person variation was still estimable because of the repeated cycles.

Unfortunately, not all nutrients have adequate reference tools such as recovery biomarkers (43). This is another potential weakness of our study that is shared by other validation studies of dietary assessment tools. It is therefore possible that the Oxford WebQ performs better or worse for other nutrients than those we were able to validate it against, particularly those derived from episodically consumed foods, for which 24-hour recalls are not well-suited. Where this is particularly important, combination with other dietary assessment tools is recommended (9, 44–47).

Our measurement error models also only considered one error-prone variable at a time. In the presence of additional error-prone covariates, the error structure becomes more complex and the direction of bias may change. This commonly occurs when a nutrient and total energy intake are included in the same model. We therefore present estimates for nutrient densities as well.

Internet applications such as the Oxford WebQ are potentially more accessible to some groups, such as the younger or better educated. To address this concern, our validation study included both men and women, with a spread of ages and a range of educational backgrounds. Additionally, we repeated our analyses by age, sex, and BMI. Results were broadly comparable between men and women. Results suggested that the online format was not a deterrent to the quality of reporting in older participants. Participants with higher BMI had similar attenuation factors, but correlation with the truth was worse for total sugar and total energy intake, suggesting greater person-specific bias in reporting certain food types in this group. This provides some support for taking BMI into account in measurement error models, as others have proposed (48, 49). While the Oxford WebQ was specifically developed for the UK Biobank and the Million Women Study, the wide age range used in our validation and the exploration within demographic subgroups provide a basis for its use in other large-scale prospective studies.

Our results indicate that repeat applications of the Oxford WebQ in large-scale projects such as the UK Biobank and the Million Women Study should provide high-quality dietary information, at least for intakes of total energy, protein, sugars, and potassium. The Oxford WebQ provides results broadly similar to those obtained using the more researcher-intensive and expensive-to-administer 24-hour recall delivered and coded by a trained researcher. This should facilitate additional dietary assessments repeated over time to measure long-term diet with greater precision, providing a platform for better estimates of the relationships between diet and disease.

## Supplementary Material

kwz165_Greenwood_Web_Material

## Abbreviations

BMI: body mass index
FFQ: food frequency questionnaire
MPR: multiple-pass recall
OPEN: Observing Protein and Energy Nutrition
PABA: 4-aminobenzoic acid
TEE: total energy expenditure
