# Supplementary material for: Validation of the Oxford WebQ Online 24-Hour Dietary Questionnaire Using Biomarkers
Source: Am J Epidemiol. 2019 Jul 18;188(10):1858–67. doi: 10.1093/aje/kwz165 (PMC7254925; doi:10.1093/aje/kwz165)
Supplement: kwz165_Greenwood_Web_Material [file kwz165_greenwood_web_material.docx]

**Web Appendix and Web Tables**

**Validation of the Oxford WebQ Online 24-hour Dietary Questionnaire Using Biomarkers**

Darren C. Greenwood, Laura J. Hardie, Gary S. Frost, Nisreen A. Alwan, Kathryn E. Bradbury, Michelle Carter, Paul Elliott, Charlotte E.L. Evans, Heather E. Ford, Neil Hancock, Timothy J. Key, Bette Liu, Michelle A. Morris, Umme Z. Mulla, Katerina Petropoulou, Gregory D.M. Potter, Elio Riboli, Heather Young, Petra A. Wark, and Janet E. Cade.

- Web Appendix 1 - Sample size requirements
- Web Appendix 1 - Adjustment for PABA recovery
- References to web appendices
- Web Tables 1–4

**Web Appendix 1**

**Sample size requirements**

The recruitment target was 200 participants with complete information collected, based on similar data presented in the OPEN study and EPIC Norfolk(1,2). With this number of participants, the study would be able to estimate the attenuation factor for protein intake to a precision of approximately +- 0.08 and estimate the correlation between the Oxford WebQ and true intake (longer-term) to a precision of approximately +- 0.1. The mean difference in nitrogen intake between Oxford WebQ and MPR would be estimable to a precision of approximately +- 0.4 g.

The 160 participants included in results was 40 (20%) lower than the number specified in the sample size calculations. Subsequent precision was therefore approximately 10% lower than anticipated, e.g. estimating the attenuation factor for protein intake to a precision of approximately +- 0.09, reflected in the confidence intervals presented in the results tables.

**Web Appendix 2**

**Adjustment for PABA recovery**

Of 440 urine samples across three cycles, 24 (5%) had PABA recovery less than 50%, 175 (40%) had PABA recovery between 50% and 85%, and 113 (26%) had PABA recovery greater than 110%. When urinary nitrogen concentrations were adjusted for completeness of urine samples, and samples with PABA recovery less than 50% or more than 110% excluded, estimated protein intakes from the urinary nitrogen biomarker were higher (78g compared to 70g) and closer to the self-reported intakes, but estimates of the attenuation factor (0.24 compared to 0.27) and correlation between self-report intakes and true intake were essentially unchanged (0.42 compared to 0.40). Similarly, estimated potassium intakes were higher (2.4g vs 2.1g), but with similar attenuation factors (0.29 vs 0.31) and correlations with estimated true intake (0.39 vs 0.34).

**References to web appendices**

1. Kipnis V, Subar AF, Midthune D, et al. Structure of dietary measurement error: Results of the OPEN Biomarker Study. *Am J Epidemiol*. 2003;158(1):14-21.
2. Bingham S, Luben R, Welch A, et al. Epidemiologic Assessment of Sugars Consumption Using Biomarkers: Comparisons of Obese and Nonobese Individuals in the European Prospective Investigation of Cancer Norfolk. *Cancer Epidemiol Biomarkers Prev*. 2007;16(8):1651-1654.

**Web Table 1.**

Web Table 1. Measurement error structure for protein, potassium and total sugar intake and density as assessed by Oxford WebQ and interviewer-based 24-hour recall.^a^

|  | Variance of true intake (95% CI)  (σ*_T_*^2^) | Dietary assessment tool | Slope in regression of reported intake on true intake (95% CI)  (β*_Q_*_1 or_ β*_F_*_1_) | Variance of person-specific bias (95% CI)  (σ*_r_*^2^ _or_ σ*_s_*^2^) | Correlation between person-specific biases (95% CI)  (ρ_(r,s)_) | Variance of within-person error (95% CI)  (σ_ε_^2^ _or_ σ*_u_*^2^) |
| --- | --- | --- | --- | --- | --- | --- |
| *Nutrient intake*: |  |  |  |  |  |  |
| Protein (g) | 0.09 (0.07, 0.13) | Oxford WebQ  MPR | 0.60 (0.39, 0.80)  0.65 (0.47, 0.83) | 0.06 (0.04, 0.09)  0.03 (0.01, 0.05) | 0.72 (0.44, 1.01) | 0.11 (0.10, 0.13)  0.11 (0.10, 0.13) |
| Potassium (g) | 0.15 (0.11, 0.20) | Oxford WebQ  MPR | 0.37 (0.21, 0.52)  0.39 (0.25, 0.53) | 0.06 (0.04, 0.09)  0.04 (0.03, 0.07) | 0.71 (0.49, 0.93) | 0.10 (0.08, 0.12)  0.10 (0.09, 0.12) |
| Total sugars (g) | 0.28 (0.20, 0.39) | Oxford WebQ  MPR | 0.35 (0.20, 0.50)  0.15 (0.01, 0.29) | 0.08 (0.05, 0.13)  0.10 (0.07, 0.14) | 1.00 (0.85, 1.21) | 0.20 (0.17, 0.23)  0.17 (0.14, 0.19) |
| *Energy expenditure:* |  |  |  |  |  |  |
| Total energy expenditure (MJ) | 0.08 (0.05, 0.11) | Oxford WebQ  MPR | 0.46 (0.24, 0.69)  0.44 (0.25, 0.62) | 0.05 (0.04, 0.08)  0.03 (0.02, 0.05) | 0.76 (0.56, 0.97) | 0.09 (0.08, 0.11)  0.07 (0.06, 0.08) |
| *Nutrient density* ^b^: |  |  |  |  |  |  |
| Protein (g / MJ) | 0.11 (0.08, 0.15) | Oxford WebQ  MPR | 0.25 (0.12, 0.37)  0.20 (0.07, 0.32) | 0.02 (0.01, 0.03)  0.02 (0.01, 0.04) | 0.79 (0.55, 1.03) | 0.05 (0.05, 0.06)  0.06 (0.05, 0.07) |
| Potassium (g / MJ) | 0.17 (0.12, 0.24) | Oxford WebQ  MPR | 0.16 (0.06, 0.26)  0.26 (0.14, 0.38) | 0.03 (0.02, 0.04)  0.03 (0.02, 0.05) | 0.96 (0.77, 1.15) | 0.05 (0.04, 0.06)  0.07 (0.06, 0.08) |
| Total sugars (g / MJ) | 0.29 (0.20, 0.42) | Oxford WebQ  MPR | 0.22 (0.10, 0.35)  0.11 (-0.02, 0.23) | 0.05 (0.03, 0.08)  0.07 (0.05, 0.10) | 0.90 (0.69, 1.10) | 0.14 (0.11, 0.16)  0.12 (0.10, 0.14) |

Abbreviations: CI, confidence interval; MPR, interviewer-based Multiple-Pass 24 hour dietary Recall

^a^ All dietary measures and estimates were log-transformed.

^b^ Nutrient density for protein, potassium and total sugars was expressed in grams per MJ of total energy intake

**Web Table 2.**

Web Table 2. Attenuation factors and correlation between dietary assessment tool and true intake for protein, potassium and total sugar intake and density as assessed by Oxford WebQ and interviewer-based 24-hour recall by sex.^a^

|  | Dietary assessment tool | Male (n=68) | |  | Female (n=92) | |
| --- | --- | --- | --- | --- | --- | --- |
|  |  | Attenuation factor  (95% CI) | Correlation with true intake  (95% CI) |  | Attenuation factor  (95% CI) | Correlation with true intake  (95% CI) |
| *Nutrient intake*: |  |  |  |  |  |  |
| Protein (g) | Oxford WebQ  MPR | 0.24 (0.12, 0.36)  0.32 (0.19, 0.45) | 0.38 (0.23, 0.52)  0.46 (0.34, 0.59) |  | 0.17 (0.06, 0.27)  0.20 (0.09, 0.31) | 0.33 (0.14, 0.52)  0.36 (0.19, 0.54) |
| Potassium (g) | Oxford WebQ  MPR | 0.33 (0.15, 0.51)  0.38 (0.20, 0.56) | 0.38 (0.20, 0.55)  0.40 (0.24, 0.56) |  | 0.26 (0.09, 0.43)  0.32 (0.15, 0.49) | 0.30 (0.12, 0.48)  0.36 (0.18, 0.53) |
| Total sugars (g) | Oxford WebQ  MPR | 0.33 (0.10, 0.56)  0.11 (-0.12, 0.34) | 0.29 (0.10, 0.47)  0.10 (-0.10, 0.29) |  | 0.29 (0.14, 0.43)  0.18 (0.03, 0.32) | 0.40 (0.22, 0.58)  0.24 (0.05, 0.43) |
| *Energy expenditure:* |  |  |  |  |  |  |
| Total energy expenditure (MJ) | Oxford WebQ  MPR | 0.08 (0.00, 0.17)  0.12 (0.02, 0.21) | 0.25 (0.10, 0.40)  0.30 (0.15, 0.45) |  | 0.11 (-0.04, 0.26)  0.10 (-0.04, 0.25) | 0.14 (-0.06, 0.34)  0.13 (-0.05, 0.30) |
| *Nutrient density* ^b^: |  |  |  |  |  |  |
| Protein (g / MJ) | Oxford WebQ  MPR | 0.41 (0.16, 0.67)  0.33 (0.07, 0.60) | 0.35 (0.16, 0.55)  0.27 (0.07, 0.47) |  | 0.28 (0.06, 0.50)  0.23 (0.01, 0.45) | 0.24 (0.06, 0.41)  0.20 (0.01, 0.39) |
| Potassium (g / MJ) | Oxford WebQ  MPR | 0.41 (0.11, 0.70)  0.51 (0.19, 0.84) | 0.29 (0.09, 0.48)  0.36 (0.16, 0.55) |  | 0.25 (-0.02, 0.53)  0.43 (0.16, 0.69) | 0.18 (-0.01, 0.37)  0.34 (0.15, 0.53) |
| Total sugars (g / MJ) | Oxford WebQ  MPR | 0.40 (0.09, 0.71)  0.23 (-0.07, 0.54) | 0.27 (0.07, 0.47)  0.16 (-0.04, 0.37) |  | 0.26 (0.06, 0.46)  0.07 (-0.12, 0.26) | 0.28 (0.08, 0.49)  0.09 (-0.13, 0.30) |

Abbreviations: CI, confidence interval; MPR, interviewer-based Multiple-Pass 24 hour dietary Recall

^a^ All dietary measures and estimates were log-transformed.

^b^ Nutrient density for protein, potassium and total sugars was expressed in grams per MJ of total energy intake.

**Web Table 3.**

Web Table 3. Attenuation factors and correlation between dietary assessment tool and true intake for protein, potassium and total sugar intake and density as assessed by Oxford WebQ and interviewer-based 24-hour recall by age group.^a^

|  | Dietary assessment tool | Age <40 years (n=72) | |  | Age 40+ years (n=88) | |
| --- | --- | --- | --- | --- | --- | --- |
|  |  | Attenuation factor  (95% CI) | Correlation with true intake  (95% CI) |  | Attenuation factor  (95% CI) | Correlation with true intake  (95% CI) |
| *Nutrient intake*: |  |  |  |  |  |  |
| Protein (g) | Oxford WebQ  MPR | 0.34 (0.20, 0.47)  0.41 (0.27, 0.54) | 0.49 (0.32, 0.66)  0.58 (0.43, 0.72) |  | 0.18 (0.06, 0.30)  0.22 (0.09, 0.34) | 0.28 (0.10, 0.46)  0.30 (0.15, 0.45) |
| Potassium (g) | Oxford WebQ  MPR | 0.23 (0.02, 0.44)  0.40 (0.20, 0.60) | 0.24 (0.03, 0.45)  0.42 (0.24, 0.61) |  | 0.35 (0.21, 0.48)  0.30 (0.15, 0.45) | 0.43 (0.29, 0.60)  0.34 (0.19, 0.49) |
| Total sugars (g) | Oxford WebQ  MPR | 0.29 (0.13, 0.44)  0.17 (0.01, 0.32) | 0.45 (0.23, 0.66)  0.27 (0.04, 0.50) |  | 0.27 (0.11, 0.44)  0.09 (-0.07, 0.25) | 0.27 (0.12, 0.42)  0.08 (-0.07, 0.24) |
| *Energy expenditure:* |  |  |  |  |  |  |
| Total energy expenditure (MJ) | Oxford WebQ  MPR | 0.09 (-0.07, 0.26)  0.25 (0.09, 0.42) | 0.13 (-0. 01, 0.37)  0.32 (0.13, 0.52) |  | 0.31 (0.18, 0.44)  0.21 (0.08, 0.35) | 0.47 (0.31, 0.63)  0.31 (0.15, 0.47) |
| *Nutrient density* ^b^: |  |  |  |  |  |  |
| Protein (g / MJ) | Oxford WebQ  MPR | 0.36 (0.13, 0.60)  0.39 (0.16, 0.62) | 0.36 (0.15, 0.57)  0.41 (0.20, 0.62) |  | 0.31 (0.10, 0.53)  0.13 (-0.09, 0.36) | 0.25 (0.08, 0.42)  0.10 (-0.07, 0.27) |
| Potassium (g / MJ) | Oxford WebQ  MPR | 0.27 (-0.06, 0.60)  0.67 (0.36, 0.97) | 0.19 (-0.04, 0.43)  0.51 (0.31, 0.71) |  | 0.39 (0.11, 0.67)  0.36 (0.07, 0.65) | 0.27 (0.08, 0.47)  0.27 (0.05, 0.50) |
| Total sugars (g / MJ) | Oxford WebQ  MPR | 0.27 (0.06, 0.47)  0.03 (-0.17, 0.22) | 0.38 (0.10, 0.65)  0.04 (-0.25, 0.33) |  | 0.33 (0.09, 0.57)  0.21 (-0.02, 0.44) | 0.23 (0.07, 0.39)  0.15 (-0.01, 0.31) |

Abbreviations: CI, confidence interval; MPR, interviewer-based Multiple-Pass 24 hour dietary Recall

^a^ All dietary measures and estimates were log-transformed.

^b^ Nutrient density for protein, potassium and total sugars was expressed in grams per MJ of total energy intake.

**Web Table 4.**

Web Table 4. Attenuation factors and correlation between dietary assessment tool and true intake for protein, potassium and total sugar intake and density as assessed by Oxford WebQ and interviewer-based 24-hour recall by body mass index.^a^

|  | Dietary assessment tool | Body mass index < 25 kg/m^2^ (n=83) | |  | Body mass index 25+ kg/m^2^ (n=77) | |
| --- | --- | --- | --- | --- | --- | --- |
|  |  | Attenuation factor  (95% CI) | Correlation with true intake  (95% CI) |  | Attenuation factor  (95% CI) | Correlation with true intake  (95% CI) |
| *Nutrient intake*: |  |  |  |  |  |  |
| Protein (g) | Oxford WebQ  MPR | 0.28 (0.16, 0.41)  0.32 (0.19, 0.45) | 0.41 (0.25, 0.58)  0.44 (0.30, 0.59) |  | 0.25 (0.12, 0.38)  0.35 (0.21, 0.49) | 0.38 (0.20, 0.57)  0.48 (0.32, 0.64) |
| Potassium (g) | Oxford WebQ  MPR | 0.35 (0.16, 0.55)  0.41 (0.21, 0.61) | 0.35 (0.17, 0.52)  0.39 (0.23, 0.55) |  | 0.26 (0.09, 0.42)  0.30 (0.12, 0.47) | 0.33 (0.13, 0.52)  0.35 (0.16, 0.54) |
| Total sugars (g) | Oxford WebQ  MPR | 0.30 (0.15, 0.45)  0.18 (0.03, 0.32) | 0.44 (0.25, 0.64)  0.26 (0.06, 0.46) |  | 0.30 (0.08, 0.53)  0.08 (-0.14, 0.31) | 0.26 (0.08, 0.45)  0.07 (-0.12, 0.26) |
| *Energy expenditure:* |  |  |  |  |  |  |
| Total energy expenditure (MJ) | Oxford WebQ  MPR | 0.24 (0.10, 0.38)  0.29 (0.16, 0.43) | 0.36 (0.17, 0.56)  0.42 (0.26, 0.58) |  | 0.20 (0.05, 0.35)  0.19 (0.03, 0.35) | 0.28 (0.08, 0.48)  0.24 (0.05, 0.43) |
| *Nutrient density* ^b^: |  |  |  |  |  |  |
| Protein (g / MJ) | Oxford WebQ  MPR | 0.45 (0.22, 0.68)  0.41 (0.17, 0.64) | 0.38 (0.21, 0.56)  0.33 (0.16, 0.51) |  | 0.22 (-0.01, 0.46)  0.14 (-0.10, 0.38) | 0.19 (0.00, 0.39)  0.12 (-0.09, 0.33) |
| Potassium (g / MJ) | Oxford WebQ  MPR | 0.43 (0.11, 0.74)  0.71 (0.40, 1.01) | 0.27 (0.08, 0.46)  0.47 (0.30, 0.64) |  | 0.23 (-0.06, 0.51)  0.27 (-0.01, 0.55) | 0.18 (-0.04, 0.39)  0.23 (0.00, 0.46) |
| Total sugars (g / MJ) | Oxford WebQ  MPR | 0.24 (0.05, 0.43)  0.08 (-0.09, 0.26) | 0.33 (0.09 0.58)  0.13 (-0.14, 0.40) |  | 0.37 (0.07, 0.68)  0.19 (-0.11, 0.49) | 0.25 (0.05, 0.44)  0.12 (-0.07, 0.31) |

Abbreviations: CI, confidence interval; MPR, interviewer-based Multiple-Pass 24 hour dietary Recall

^a^ All dietary measures and estimates were log-transformed.

^b^ Nutrient density for protein, potassium and total sugars was expressed in grams per MJ of total energy intake.
